# Research methods challenges: A case study of preparedness in The Bahamas

**DOI:** 10.4102/jamba.v16i1.1565

**Published:** 2024-02-07

**Authors:** Trevor O. Johnson, Jessica Jensen

**Affiliations:** 1 Department of Emergency and Disaster Science, Faculty of Social Sciences, North Dakota State University, Fargo, United States; 2 Department of Global Operations, Pacific Disaster Center, Kihei, United States; 3 Country of Bahamas Office, InterAmerican Development Bank, Nassau, Bahamas; 4 RAND Corporation, Department of Defense and Political Sciences, Santa Monica, United States

**Keywords:** The Bahamas, developing countries, preparedness, research, methodological

## Abstract

Disaster research is essential for developing more robust and contextualised policies. It is, therefore, no surprise that multilateral organisations like the United Nations and the World Bank have called for enhanced disaster-related frameworks, legislation and policies in developing countries using quality data. However, internal and external researchers and practitioners often face significant challenges collecting data in these nations because of a range of problems including, but not limited to, incomplete sampling frames, inadequate infrastructure or unstable governments. This reality leads one to question: is the cart coming before the horse? This study explored individual and household (IH) preparedness in The Bahamas – a small island developing state in the Caribbean. An online survey was used, and 629 Bahamians opted to participate. However, the researchers faced many barriers to collecting representative data. This case study, therefore, discusses the range of methodological challenges faced by the researchers and their impact on this study.

**Contribution:**

This article substantially contributes to the disaster literature by exploring the challenges associated with conducting IH preparedness research in The Bahamas. This article also reminds practitioners and academics of the issues associated with collecting data in developing nations and its implications for policy enhancement and development. Furthermore, the authors present various recommendations ranging from enhanced funding to recognising the need for methodological innovation to support continuous research in countries like The Bahamas.

## Introduction

No community, jurisdiction or nation is immune from hazard events (Ronan, Crellin & Johnston 2012). According to the 2020 World Disaster Report, the number of disasters has increased by 35% since the 1990s (International Federation of Red Cross 2020). In 2019, various hazard events impacted 97 million persons and claimed more than 27 000 lives globally. Most of these deaths occurred in the developing and least-developed world (International Federation of Red Cross 2020). The Bahamas, a developing country, was recently ravaged by Hurricane Dorian – the deadliest and costliest storm in its history (Johnson 2020). The impact of disasters like Hurricane Dorian and the negative experiences of communities in the post-disaster context make preparedness an essential function (Atreya et al. 2017; Bollettino et al. 2020; Kirschenbaum 2006).

**FIGURE 1:**
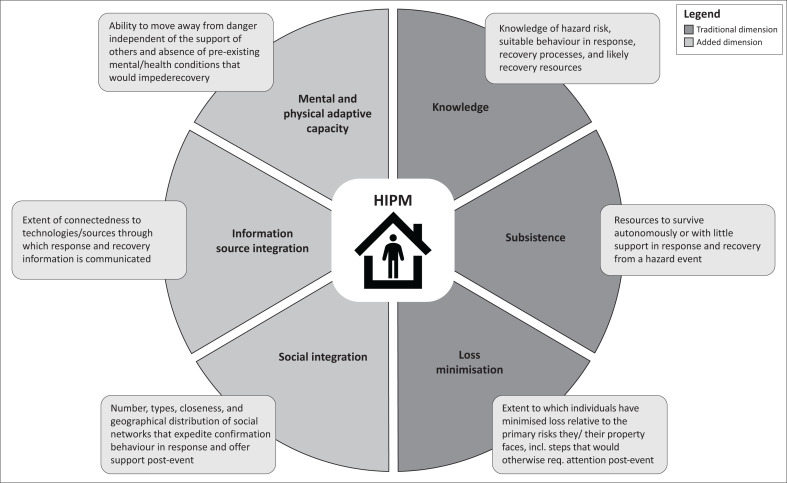
Overview of the Holistic Individual Preparedness Model.

The Sendai Framework for Disaster Risk Reduction, a document reflecting global consensus regarding what causes hazard events and what should be done around the world to address them, has called for broad stakeholder involvement in all phases of disaster management, especially preparedness (United Nations Office for Disaster Risk Reduction 2015). Despite preparedness being encouraged, individuals and households (IHs) continue to suffer in the post-disaster context for various reasons. Many attribute these experiences to a lack of readiness (Abramson et al. 2010; Atreya et al. 2017). This reality has resulted in researchers studying the barriers and facilitators of IH preparedness for decades (e.g. Edwards 1993; Farley et al. 1993; Wang et al. 2021). However, quantitative and qualitative IH preparedness studies are plagued with theoretical and measurement problems that render them of uncertain value (e.g. Rampengan et al. 2014; Shah et al. 2017). Throughout the literature, scholars rarely define preparedness despite it being their study’s dependent variable or keyword (e.g. Edwards 1993; Farley et al. 1993; Najafi et al. 2015; Nakagawa 2017; Oloke 2021; Spittal et al. 2008). Further to this, there are many challenges associated with the measurement of IH preparedness in the literature. Examining the items used to measure preparedness reveals many idiosyncrasies as scholars use entirely different things in their inventories of IH preparedness. Chan et al. (2014) measured preparedness with eight things: food supplies, drinking water, essential medication, long-term medication, first aid kit, backup electric stove, fire extinguisher and anti-flooding leaking measures. Conversely, Kirschenbaum (2006) used a four-item scale that included these measures: (1) levels of provisions or supplies available in the home, (2) knowledge of and ability to utilise survival and first aid skills, (3) having evacuation and family plans at the ready and (4) protective physical shelters or sealed room. Despite investigating the same concept, these two examples present stark differences in what preparedness looks like. While the Bahamian context warrants an examination of IH preparedness, this research must not replicate the theoretical problems of prior research. Therefore, this study leveraged the Holistic Individual Preparedness Model (HIPM) – a new conceptual framework that addresses many theoretical issues underlying IH preparedness research. The HIPM clearly defines preparedness and comprises dimensions that should be considered to measure preparedness (Johnson & Jensen 2023). The six dimensions or areas of preparedness are: knowledge, subsistence, loss minimisation, social integration, information integration, and mental and physical adaptive capacity. The HIPM recognises that preparedness is dynamic, context-dependent, multidimensional, and informed by what makes people better respond and recover from disasters. Refer to Figure 1 for a brief overview of the HIPM.

In the developing context, research in this topical area is a relatively new focus (e.g. Pacheco, Pereira & Rego 2021; Sadeka et al. 2020). In The Bahamas, research on IH preparedness is absent. Hence, this study explored the following research question:

1. To what extent are Bahamians prepared for disasters?

The Bahamas is an archipelagic nation comprised of 700 islands and 200 cays, rocks and islets (ECLAC 2011). Approximately 19 of these islands and cays are inhabited (ECLAC 2011). The country extends over 100 000 square miles across the Atlantic Ocean (Neely 2012). The country’s total landmass is 5380 square miles, smaller than the state of Massachusetts (US Army Corps of Engineers 2004). The country’s population is approximately 400 000 people (Hoogeveen & Pape 2020; United Nations Population Fund 2022). More than 65% of the country (*n* = 265 000) resides in the capital, Nassau, New Providence (United Nations 2020). All Family Islands have fewer than 50 000 people (Government of The Bahamas 2018; United Nations 2020). Ragged Island, the country’s least-populated island, has a population of 72 people (United Nations 2020).

The Bahamas’ gross domestic product (GDP) is about $11.25 billion (United Nations 2020). The GDP per capita is approximately $28 600.00 (United Nations 2020). Tourism and banking are the main economic drivers (ECLAC 2011). These industries have made The Bahamas the wealthiest country in The Caribbean (ECLAC 2011). However, the Bahamian landscape is confronted with various hazards and vulnerabilities that threaten its economic potential (Carrera Marquis, Franklin & Espiga 2022; Rhiney 2015).

The Bahamas’ geographic positioning and climatic conditions make the country vulnerable to hurricanes, bushfires and localised flooding (NEMA-Bahamas 2020). The country is squarely situated in the ‘hurricane belt’ (Deopersad et al. 2019:13). The hurricane belt is the portion of the Atlantic Ocean that sees periods of enhanced tropical cyclone activity each year (Carrera Marquis et al. 2022). Neely (2012) noted that five of the six most hurricane-impacted Caribbean islands are a part of the Bahamian archipelago (i.e. Abaco, Grand Bahama, Bimini, New Providence and San Salvador). The costliest disasters in the country’s history have occurred over the past decade – Hurricanes Joaquin, Matthew, Irma and Dorian (Carrera-Marquis et al. 2022; Johnson 2020). The losses and damages of those events were more than $USD 4.2 billion (Carrera-Marquis et al. 2022; Johnson 2020). Table 1 summarises the notable hazard events the country has faced over the past century.

**TABLE 1:** Hurricanes to impact The Bahamas since 1932.

Hazard event	Year	Category	Region impacted
The Great Bahamian Hurricane of 1932	1932	5	Entire archipelago
Hurricane Donna	1960	4	South-eastern islands
Hurricane Betsy	1965	4	North-western islands
Hurricane Andrew	1992	4	Central and Extreme North-western islands
Hurricane Floyd	1999	4	Central and North-western islands
Hurricane Francis	2004	4	Entire archipelago
Hurricane Jeanne	2004	2	Extreme North-western islands
Hurricane Wilma	2004	3	North-western islands
Hurricane Ike	2008	4	South-eastern islands
Hurricane Irene	2011	3	Entire archipelago
Hurricane Joaquin	2015	5	South-eastern islands
Hurricane Matthew	2016	4	Central North-western islands
Hurricane Irma	2017	4	South-eastern islands
Hurricane Dorian	2019	5	Extreme North-western islands

*Source*: Neely, W., 2012, *The great Bahamian hurricanes of 1899 and 1932*, iUniverse, Bloomington; Carrera-Marquis, D., Espiga, F. & Johnson, T., 2022, *IDB Dala assessments: Synthesis: Joaquin, Matthew, Irma, and Dorian*, Inter-American Development Bank, Nassau

The consistent impacts of various hazards make The Bahamas a high-risk country. The hazardous nature of The Bahamas, combined with its physical, social and economic vulnerabilities, increases the impacts, and needs, associated with hazard events (Rhiney 2015; Thomas, Nicolls & Finlayson 2021). With regards to the country’s physical vulnerability, its low-lying nature is of great concern (Government of The Bahamas 2015). More than 80% of the country’s landmass is within 5 feet of mean sea level (Johnson 2020). Como Hill stands at 206 feet above sea level and is the highest point in the country (US Army Corps of Engineers 2004). In addition to being relatively flat, The Bahamas has limited freshwater resources (Thomas et al. 2021; US Army Corps of Engineers 2004). The Bahamas has made substantial strides regarding access to government resources and services (ECLAC 2011). However, various equity issues across the Family Islands enhance the country’s vulnerability (Government of The Bahamas 2018; United Nations 2020). Access to most government agencies and departments is limited (United Nations 2020). Moreover, The Bahamas is ranked as a middle-income nation; however, the undiversified nature of the country’s economy is a significant challenge (ECLAC 2011). Tourism, the ‘lifeblood’ of the country, accounts for more than half of the GDP (Government of The Bahamas 2018). These vulnerabilities of The Bahamas require all stakeholders to consider climate adaptation (Government of The Bahamas 2018). Thomas et al. (2021) explained that disaster preparedness is a critical adaptation measure given the reality of stronger weather events.

Despite the significance of studying preparedness in the Bahamian context given the vulnerabilities of the country, the researchers could not fully explore this topical area in The Bahamas. Specifically, the researchers encountered several methodological problems, resulting in the collection of skewed and non-normal data. Like this study, many IH preparedness studies employed convenience sampling because of issues like incomplete sampling frames, lack of digitisation, data privatisation, inadequate telecommunication infrastructure, poor quality roads or hostile central government representatives (e.g. Alfaro-Alejo et al. 2021; Appleby-Arnold et al. 2018; Dollete 2020). The nature of the data and the sampling method had implications on the extent to which: (1) conclusions were made about Bahamian preparedness; and (2) recommendations were given to the Bahamian government.

Scholars have written about the issues associated with collecting data in the developing context (e.g. Elahi 2008; Mennecke & West 2001; Van Niekerk, Coetzee & Nemakonde 2020; Wamsler & Johannessen 2020). Similarly, multilateral organisations have explored challenges associated with conducting research in developing and least-developed nations (e.g. Hoogeveen & Pape 2020; UN DESA & UNDRR 2022). Simultaneous to identifying these issues in academia and practice, there have been increasing calls for countries like The Bahamas to develop, enhance and refine disaster risk management policies (Hoogeveen & Pape 2020; UN DESA & UNDRR 2022). However, these policies should be dependent on quality research (Elahi 2008; Hoogeveen & Pape 2020; Jensen 2011; Mennecke & West 2001; UN DESA & UNDRR 2022; Van Niekerk et al. 2020; Wamsler & Johannessen 2020). Here lies the conundrum – how do these countries develop policies if there are significant gaps in collecting quality data? Instead of presenting the preparedness data associated with this study, this article will highlight the challenges the researchers encountered with hopes of raising awareness of this vexing issue in the Bahamian landscape and the developing world. Of note, this study is not suggesting that this problem is only relevant to the developing world, as several authors have brought this issue to the fore in the context of developed countries (e.g. Jensen 2011; Phillips 2005, 2014). However, this study focusses on developing countries because of their increasing: (1) vulnerabilities to climate change; and (2) deaths, losses and damages associated with hazard events in recent years.

## Case study: Preparedness in The Bahamas

UN DESA and UNDRR (2022) noted the need for enhanced information to be collected to understand the vulnerabilities and capacities of communities in small island developing states (SIDS). Hoogeveen and Pape (2020) and Van Niekerk et al. (2020) posited that this quality data is essential for meeting the goals and targets of the Sendai Framework. Like many SIDS, the Bahamas has many policy gaps in disaster management (Deopersad et al. 2019). Therefore, this study sought to explore the topical area with hopes of filling some of these gaps. Data were collected with an online survey instrument. The six dimensions of preparedness were operationalised to measure IH preparedness. Convenience sampling was employed, and when data collection ceased, 629 Bahamians opted to participate. The results of this study suggest that the participants’: (1) information integration was significantly lower than the other dimensions, (2) income was positively related to preparedness dimensions and (3) knowledge and information integration leaned most towards the items that were relevant to disaster response. However, this study faced many challenges that rendered these findings invalid with respect to the wider Bahamian population.

### Methodological steps taken to explore preparedness in The Bahamas

It is essential to discuss the extensive efforts taken by the researchers to collect quality data. The population for this study comprised all Bahamians. The estimated population of The Bahamas is 400 000 (Hoogeveen & Pape 2020; United Nations Population Fund 2022). Conducting a probabilistic sample in this context was difficult and practically not possible. Probability sampling occurs when all units are equally likely to be selected (Blackstone 2019; Chambliss & Schutt 2018; Creswell 2014; Maxim 1999; Stockermer 2019). However, The Bahamas does not have a developed mailing or postal system. Attempting to conduct this study with a smaller subset of the population (e.g. an island) would face similar problems because of the lack of an organised mailing system. Further, random digit dialling or purchasing a random email panel is unavailable in the Bahamian context – the local researcher contacted all major telecommunication companies for support in this regard. Moreover, any random sample would only be feasible within a very small subset of the population (e.g. teachers in a school), rendering the findings of minimal practical value.

Considering these challenges, this study employed a non-probability sampling method – convenience sampling. Stockermer (2019) explained that while a random sample is ideal for quantitative research, investigators must balance rigour and practicality. Convenience sampling occurs when elements are selected because they are available or easily accessible (Bhattacherjee 2012; Creswell 2014; Phillips 2014; Stockermer 2019). Baxter, Courage and Caine (2015) suggested that researchers engaged in convenience sampling should ensure that the data is as contextual as possible. Contextualisation considers reaching as many units as possible in the population of interest. Therefore, this study attempted to reach as many Bahamians as possible. Furthermore, electronic surveys are most appropriate and feasible when considering: (1) the context of The Bahamas, and (2) the data collection method of prior investigations in the country (e.g. Saunders 2011, 2020). This study, therefore, employed an electronic web survey for data collection (i.e. SurveyMonkey).

The Bahamas is a unique context requiring novel approaches to collecting data. Internet access is available across all islands of The Bahamas (Government of The Bahamas 2018). Bahamians are significantly engaged in Facebook for various reasons (Dwivedi, Yadav & Venkatesh 2011; Saunders 2011, 2020). Therefore, this study used: (1) media and government agencies, and (2) social media to aid in recruiting participants. The media and government agencies were asked to post the opportunity to participate in this study three times per week for 3 weeks on their Facebook pages in November 2022. These partners agreed to use the survey link, narrative and visuals provided. These six agencies (The Office of The Prime Minister, The National Emergency Management Agency [NEMA], The Ministry of Youth Sports and Culture, EyeWitness News, Our News and ZNS News) were approached based on their social media following and credibility. The social media following of these partners is critical to ensuring that as many Bahamians as possible would participate in this study.

Three of the six organisations that the local researcher approached represented government agencies (i.e. The Office of The Prime Minister, the NEMA and the Ministry of Youth Sports and Culture, and the other three represent the media (i.e. EyeWitness News, Our News and ZNS News). The NEMA, the Ministry of Youth Sports and Culture, EyeWitness News and Our News agreed to partner in this project. Table 2 summarises this information.

**TABLE 2:** Data collection partners.

Agency	Following as of 17 October 2022	Date of approval
The Office of The Prime Minister	71 000	No response
The National Emergency Management Agency	21 000	16 May 2022
The Ministry of Youth Sports and Culture	7400	20 May 2022
EyeWitness News	121 637	05 May 2022
Our News	88 087	10 May 2022
ZNS News	58 000	No response

These agencies were to serve as the primary source for recruiting participants. However, only two of the agencies (i.e. NEMA and Our News) followed the procedures regarding the frequency of posting. In light of this, the local researcher requested the participation of his network via social media. Further, the researcher contacted another government institution (i.e. the Department of Local Government) to assist with recruiting participants. However, this path did not result in significant support. The local researcher then contacted the Rotary Clubs, a pervasive non-profit organisation in The Bahamas, to gather the support of its members and followers to participate in this research using the same marketing material given to the initial partners. Rotary partners agreed to assist, and various board members noted advertising the survey on social media platforms.

After 3 weeks of posting by research partners and the local researcher, 592 Bahamians completed the survey. However, the researchers were not pleased with the distribution of the data nor the number of completions. Therefore, a Facebook page was created (i.e. Disaster Preparedness in The Bahamas). The purpose of this page was to create a boosted post to reach as many Bahamians as possible. This post was promoted for 5 days and received 30 000 views. However, only an additional 39 Bahamians completed the survey. As of 05 December 2022, 990 Bahamians followed the survey link when data collection ceased. Interestingly, 349 Bahamians: (1) agreed to participate, and (2) were over the age of 18, but did not answer a single substantive question. In other words, 35% of Bahamians that clicked the link did not answer a single question. It is unclear why participants stopped. The rationale for the lack of effort to begin a substantive question could not be about survey length, as the questions appeared one at a time. Of the 641 Bahamians who completed the survey, five affirmatively declined participation, and seven were under the age of 18. This resulted in a sample size of 629. The completion rate was 63.5% (629 of 990).

Undoubtedly, this study undertook many steps to collect meaningful data. From using government agencies and media outlets to creating advertisements on social media, the researchers tried their best to meet the goal of this study, given budgetary constraints. Despite these efforts, the sample was not representative of the Bahamian population. The subsequent section will describe the sample of this study.

### The sample

The survey was designed to gather data on Bahamians – the unit of analysis. Analysis of basic demographics revealed that 73.6% of the participants were female (*n* = 463), and 72.3% were under the age of 45 (*n* = 452). Their ages ranged from 19 to 95 years. University education as their highest level of education was noted by 69.0% (*n* = 436) of the participants. Most of the participants resided on the island of New Providence (*n* = 288, 45.8%). Grand Bahamians (*n* = 271, 43.1%) and Abaconians (*n* = 35, 5.6%) represented the second and third-highest number of participants, respectively. The income of the participants varied; however, 40.9% selected the category indicating that they earned between $20 001.00 and $40 000.00 (*n* = 271). Notably, 69.0% of the participants earned less than $40 000.00 (*n* = 439). The number of people living in a house ranged from one to eight, or more. Household size was bimodal. All 146 (23%) participants indicated they had two persons in their homes, and the same number of participants noted their household size as four. Furthermore, 56.0% (*n* = 317) of the participants had dependents in their homes, and 67.7% (*n* = 426) of the participants owned their homes.

Because of the fact that this study employed a convenience sample, it is important to compare its demographics with that of the wider population. The country’s most recent census was used (Department of Statistics 2012). Department of Statistics (Bahamas) (2012) outlined that the proportion of Bahamians with a university education was 16.1%. However, 69.3% of Bahamians in this sample had a university education. This example highlights the unrepresentative nature of this sample. Table 3 highlights other points of comparison that were used. The most striking differences are highlighted in grey.

**TABLE 3:** Comparison of the sample to the population.

Demographic variable	Point of comparison	Census 2010	This study
Age	Median age	29.4 years	32 years
Sex	Proportion of male	48.44%	26.4%
	Proportion of female	51.56%	73.6%
Island of residence	Proportion residing in New Providence	70%	45.8%
	Proportion residing in Grand Bahama	15%	43.1%
	Proportion of residing in Family Islands	5%	11.1%
Highest level of education	Proportion with a University Education	16.1%	69.3%
Annual income	Mode	$20 001–$40 000	$20 001–$40 000
Household size	Mode	1	2 and 4
Dependents in home	Mode	Yes	Yes
Homeownership	Proportion owned	58.9%	67.7%

The distribution of the sample obtained in this study is not representative of The Bahamas along many demographic variables (i.e. sex, island of residence, highest level of education, household size and homeownership). The original intent of this study was to conduct multiple linear regression. However, this test requires a series of assumptions to be met (Blackstone 2019; Blaikie 2011; Jaccard & Jacoby 2020; Stockermer 2019). The data did not meet these assumptions, limiting this study’s approach to data analysis. Further, the non-representative nature of the data meant that the results were not generalisable.

### Ethical considerations

Ethical clearance to conduct this study was obtained from the North Dakota State University, Research Integrity and Compliance department (No. IRB0004552).

### Research culture

While sampling was a significant issue, additional challenges were faced because of the research culture of The Bahamas. To remind the reader, this study undertook extensive efforts to ensure maximum participation:

1. Several organisations posted the survey link and narrative on their social media accounts (i.e. NEMA, the Ministry of Youth, Sports, and Culture, Our News, EyeWitness News, and Rotaract District 7020).
2. Various media outlets interviewed the local researcher about the survey on three separate occasions.
3. A Facebook page for this research project and advertisements were paid for.
4. The local researcher shared the survey link and narrative via his networks and social media platforms.

Given this effort, participation in this survey was less than expected. Participation issues become apparent when considering that 349 Bahamians agreed to participate in the study and were over 18 years, but did not answer a single substantive question. In other words, they quit without starting. Further, well in excess of over 30 000 persons viewed sponsored social media posts (i.e. ads) about this survey. But only a tiny fraction of those persons opted to participate in this study. This trend could reflect the unwillingness of some persons to participate in survey research.

Further, some of those who participated in this project complained about the survey’s length. This concern appeared during the pilot study and is part of the reason why the survey instrument was not assessed with split-half reliability. In addition, various independent variables (IVs) were removed from the survey instrument because of concerns about survey length (i.e. religiosity). Despite these considerations, participants who knew the local researcher personally texted or called *ad nauseum* to share their concerns about the post-piloted survey instrument. See two comments that were communicated to the local researcher.

1. ‘*[Local reseacher’s name]*, you is my boy, but this survey is too long; this is more than what the government collects for the census.’
2. ‘I literally wanted to give up while doing this; I had to get a meal to give me the strength to complete.’

The concern of the participants about survey length made the researchers question whether some of the participants who completed the survey did so accurately and meaningfully or if, because of length, they may have ‘pencil whipped’ the survey. Of note, these findings with respect to the research culture of the country are merely conjectures. But they are worth considering in the context of what was consistently observed of the participants during this project. If these claims are true about the country’s research culture and sampling issues continue, the quality of social science research and the development of contextualised and grounded policy in The Bahamas will be significantly threatened.

## Discussion

Individual and household (IH) preparedness is crucial in the Bahamian landscape. This study aimed to identify the dimension(s) or areas where Bahamians were more or less prepared. Furthermore, this study intended to make meaningful conclusions about total preparedness, and identify the factors that explain variation in the preparedness of Bahamians. This study wanted to obtain these data to give constructive advice to disaster management institutions about IH preparedness to inform their policy and training initiatives. For example, the participants least affirmed the building construction measures not listed in the country’s building codes (i.e. hurricane-proof windows, installed shutters, and base elevation above flood level). This finding could have resulted in recommendations for updating the country’s building codes. However, this suggestion cannot be made because of the range of problems mentioned in prior sections. These challenges do not mean that research should not occur in The Bahamas. There is overwhelming support for the opposite, in that enhanced research is needed in countries like The Bahamas (e.g. Elahi 2008; Hoogeveen & Pape 2020; Mennecke & West 2001; UN DESA & UNDRR 2022; Van Niekerk et al. 2020; Wamsler & Johannessen 2020).

Obtaining a random sample of the general population over the age of 18 years appears to be impossible, given the context of The Bahamas. Any robust quantitative study requires avenues to gather a random sample to ensure that its results can be: (1) generalised, and (2) relevant to policy. However, this study faced tremendous difficulties in obtaining a random sample. Various means to obtain a random sample (e.g. mailed surveys, email panels or random digit dialling) were known. However, these methods for data collection were not possible in the Bahamian context. The country does not have a codified mailing system. Furthermore, this study explored many avenues in an attempt to obtain a comprehensive listing of Bahamians with their relevant contact information. This effort did not yield any favourable responses. These challenges resulted in a convenience sample being conducted. As a result, the sample, once gathered, was not consistent with the country’s most recent census. The sample mainly comprises females, Grand Bahamians, and those with a university education. For these reasons, this study cannot generalise beyond the 629 Bahamians who participated in this study, as the sample did not represent the wider population. The researchers knew that using social media as the primary recruitment source could have excluded various demographics from participating in this study (e.g. males, Family Island participants, and persons not university educated). However, this option was most feasible when considering the country’s geographical, social and infrastructural context.

Disaster research is often cumbersome; however, it is vital to expanding knowledge bases and meeting the ambitious legislative and policy agenda set forth by various multilateral organisations (Elahi 2008; Hoogeveen & Pape 2020; Mennecke & West 2001; UN DESA & UNDRR 2022; Van Niekerk et al. 2020; Wamsler & Johannessen 2020). Jensen (2011) explained that researchers in the disaster space are sometimes interested in understanding how humans manage or cope with disasters (i.e. social science research). This study exemplifies social science research in the hazard and disaster space. This type of research is complex and requires much nuance and detail (Chambliss & Schutt 2018). The methodological difficulties associated with manoeuvring the Bahamian landscape further compounded the inherent complexities of social science research, making it difficult for the researchers to collect quality data. The challenges of obtaining quality data are not limited to survey research or social science research in the hazards and disaster space. Van Niekerk et al. (2020) and Mennecke and West (2001) explained that inadequate, and in some instances absent, data with respect to vulnerabilities, capacities, and disaster experiences make risk assessments in developing countries very difficult. Hoogeveen and Pape (2020) explained that risk assessments are fundamental to any disaster policy or plan. Moreover, UN DESA and UNDRR (2022) noted that SIDS face tremendous challenges in assessing loss and damages, risk mapping, and rapid needs assessments because of various infrastructural or capacity gaps. The ability to obtain quality data in the developing world has significant implications when considering the impacts of climate change.

Developing countries suffer the highest frequency of disasters in the world and their resilience hinges on their ability to adapt to the changing climate. Climate adaptation encompasses a range of structural and non-structural measures. Policies, frameworks, and laws are critical components of climate adaptation. However, these measures should be developed based on the findings of research. For example, a country’s Recovery Aid Assistance Program should be based on the prior recovery experiences of IHs. The serious question to be asked and answered is, how often does this occur? Some researchers argue that based on the persistent issues seen with how governments handle disasters, research is not influencing policy and decision-making as much as it should (e.g. Faber et al. 2014; Jensen 2011; Oyola-Yemaiel & Wilson 2005; Phillips 2005). There is a dire need for change! But how can this change be actualised?

Firstly, governments must recognise the importance of research in policy and decision-making. And there is no doubt that higher education is the vehicle for synergising efforts between research and policy (Jensen 2011). Higher education programmes have exponentially increased around the world since the early 2000s. In the United States, there are over 600 degree or certificate programmes (Cwiak 2014). Furthermore, numerous countries, including Canada, South Africa and Japan, have developed disaster risk management or disaster resilience programmes to enhance and fortify human capacity. Academic institutions must work in tandem with practitioners and governments. This is a cry that many stakeholders have echoed (e.g. Bartunek 2007; Jensen 2011). Bartunek (2007) recommended that the following be considered: joint symposia, academic advisory councils, executives in residence or sabbaticals in industry. Jensen (2011) explained that these measures help to reduce the tension between practitioners and academics.

Secondly, there needs to be a more significant investment by governments and multilateral organisations. Governments, and yes, those in the developing context, should place great effort in supporting researchers and investing in research infrastructure. A robust census is a good starting point. When this study was conducted, the most recent census in The Bahamas was completed in 2010 – more than 13 years old! This is highly problematic. Elahi (2008) recommended governments decentralise data entry and train or educate more statisticians to ensure that issues like inadequate sampling frames can be remedied. Moreover, multilateral organisations have repeatedly echoed the need developing countries. augment policies and enhance the quality of hazard and disaster information available. However, these institutions must equally invest in supporting the research machinery of developing countries via grants or funds – not loans.

Thirdly, researchers must be innovative when collecting data. In an ideal world, the challenges associated with collecting data in the developing world would disappear. However, when one considers the many priorities, shocks and stressors these countries face, addressing all of these data collection challenges will take considerable time. Therefore, Hoogeveen and Pape (2020) asserted that the proliferation of mobile phones and inexpensive handsets should be considered when face-to-face household surveys are changed. Additionally, these authors noted that in the absence of sampling frames, Geographic Information Systems (GIS)-based sampling approaches could be employed to estimate population density and demarcate enumeration areas. Hoogeveen and Pape (2020) also suggested that the researchers should consider using video testimonials as a cost-effective way to allow survey respondents to share their perspectives. The authors touted the successes of a project in South Sudan in which participants sent videos to researchers who were estimating poverty levels in the country.

## Conclusion

Considerable time and effort were given to ensure this study would yield valuable results. However, the researchers faced many issues in collecting quality data. Some of these issues are not unique to the Bahamian landscape, but are a common occurrence in many developing countries. Climate change requires many adaptation measures, including but not limited to legislative and policy reform. However, the success of these reforms hinges on contextualised and quality research data. This project is one example of how difficult it can be to conduct research in the developing world. This problem requires enhanced action by governments and multilateral organisations. Future agreements by various multilateral organisations must clearly recognise this issue and play a role in mending this challenge. Disaster risk management requires sea walls, stronger ecosystems, and greater coordination (among others) to be holistic and risk-reducing, but we cannot neglect the significance of research and quality data. This article sought to bring this front and centre.
